# A review on Zika virus outbreak, epidemiology, transmission and infection dynamics

**DOI:** 10.1186/s40709-020-00115-4

**Published:** 2020-03-04

**Authors:** Syeda Sidra Kazmi, Waqar Ali, Nousheen Bibi, Faisal Nouroz

**Affiliations:** 1 Department of Bioinformatics, Hazara University Mansehra, Mansehra, Pakistan; 2 Department of Botany, Hazara University Mansehra, Mansehra, Pakistan

**Keywords:** Zika virus, *Aedes* mosquito, Epidemiology, Guillain–Barré syndrome, Microcephaly, Treatment

## Abstract

Zika virus (ZIKV) is a newly emergent relative of the Flaviviridae family and linked to dengue (DENV) and Chikungunya (CHIVKV). ZIKV is one of the rising pathogens promptly surpassing geographical borders. ZIKV infection was characterized by mild disease with fever, headache, rash, arthralgia and conjunctivitis, with exceptional reports of an association with Guillain–Barre syndrome (GBS) and microcephaly. However, since the end of 2015, an increase in the number of GBS associated cases and an astonishing number of microcephaly in fetus and new-borns in Brazil have been related to ZIKV infection, raising serious worldwide public health concerns. ZIKV is transmitted by the bite of infected female mosquitoes of *Aedes* species. Clarifying such worrisome relationships is, thus, a current unavoidable goal. Here, we extensively described the current understanding of the effects of ZIKV on heath, clinical manifestation, diagnosis and treatment options based on modern, alternative and complementary medicines regarding the disease.

## Introduction

Among the family of viruses, Zika virus (ZIKV) is an emerging evolving virus on the western hemisphere, though it was initially reported from Uganda in 1940s [1, 2]. Transmission of ZIKV is related to the two other imperative arbo-viruses including dengue virus (DENV) and chikungunya virus (CHIVKV) [3]. In a quest to solve the dilemma of yellow fever, a study conducted in 1947 isolated the first novel virus from the blood of a sentinel rhesus macaque placed in the Zika Forest of Uganda [4, 5]. ZIKV stayed relatively silent for almost 70 years and all of a sudden emerged all over the America after Pacific Islands to Brazil [6]. Recently it was identified that the ZIKV strain found in the Americas had escalated to Angola and was linked with a cluster of microcephaly [7–9]. Hill et al. also reported similar results based on full virus genome analysis [9]. All the above mentioned studies endorse overview of mosquito-borne transmission of the ZIKV strain from the Americas into continental Africa. World Health Organization (WHO) declared it as emergency of public health with international concern as a result of global alarm created by ZIKV by becoming first foremost infectious disease coupled with defects of human birth revealed in more than a half of century [10].

## History and epidemiology of ZIKV

**Fig. 1:**
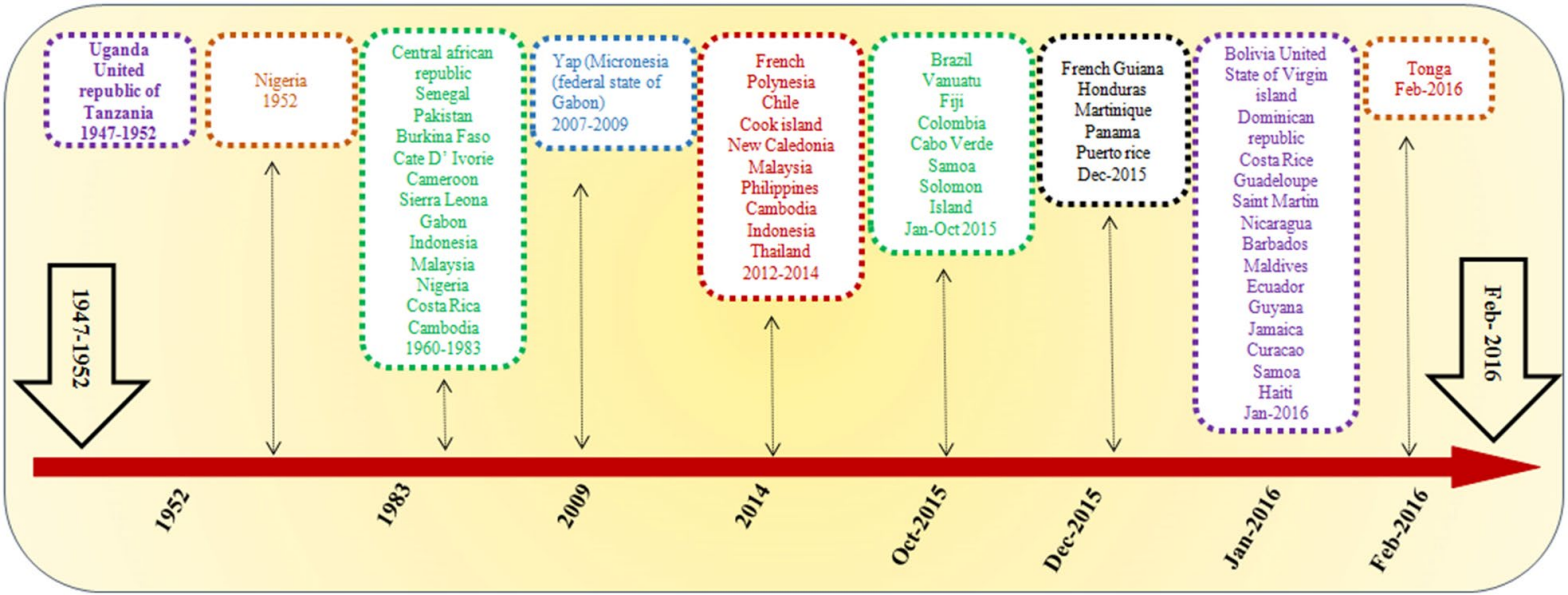
Chronological time-line of ZIKV epidemic from 1947–2016

ZIKV is a member of family Flaviviridae and spread through *Aedes* genus. Other members of this family include arboviruses, dengue virus and Japanese encephalitis viruses [11]. ZIKV antibodies were also detected in animal species, especially non-human primates [12]. ZIKV was also isolated from several mosquito species in Africa and Asia including arboreal mosquitoes as *Aedes africanus* or mosquitoes with a large tropical and subtropical distribution as *Aedes aegypti* [13] and *Aedes albopictus*, respectively [14]. Studies reported that ZIKV has three main lineages, two from Africa and one from Asia [15, 16]. The African lineage split in East and West African clusters [17, 18]. Asian lineage presents expanded geographical distribution [18], since it emerged in the Pacific Ocean [19] and South America [20, 21]. The 2015–16 epidemic occurred in the America was due to strain of the Asian lineage generally known as the American strain [22, 23]. However, some consider the American outbreak strain as its own lineage. Epidemiology studies revealed distribution of ZIKV in half of the north African continent, Vietnam, Malaysia, Indonesia, Philippines, India, Thailand and Pakistan (Fig. 1) [11, 24]. The first human case was detected in Uganda in 1952 during a study indicating the presence of neutralizing antibodies to ZIKV in sera [25]. Only few cases of infection in human were reported before 2007 when outbreak of ZIKV infection in humans occurred in Yap, Federated States of Micronesia, in the Pacific region [26]. In French Polynesia the largest epidemic of ZIKV occurred during 2013 to 2014 and extended to New Caledonia, Cook Islands, Vanuatu, Easter Island, Solomon Islands and other Pacific Islands [5]. ZIKV transmission is known in 55 countries and territories. However, only in 2015 to 2016, indigenous transmission have been reported for 41 of them, with indirect confirmation regarding circulation of virus in six countries, terminated outbreaks reported in five countries while three countries were affected with local infection [27].

## Molecular biology and virology

Flaviviridae family contains clinically important arboviruses with four genera including *Hepacivirus* (one species that is hepatitis C virus), *Pestivirus* (four species), *Pegivirus* (two species) and *Flavivirus* (53 species). Other than hepatitis C virus, most of the clinically relevant pathogens belong to the genus *Flavivirus* [28]. The most significant clinical manifestations by Flaviviruses include fever, rashes, encephalitis, visceral involvement and hemorrhagic fever [29].

**Fig. 2:**
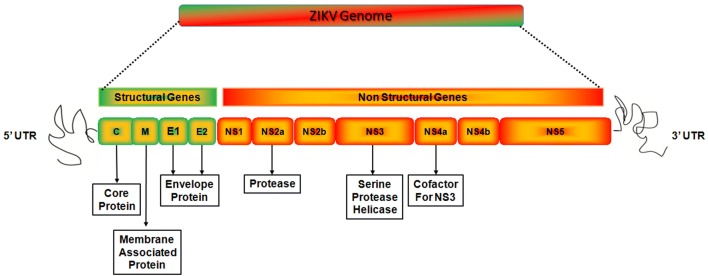
The genome organization of ZIKV

The length of ZIKV genome is 10,794 kb, comprising a positive sense single-stranded RNA molecule having two noncoding regions (NCR); 39 and 59 NCR and a long open reading frame that encode a polyprotein: 59-C-prM-E-NS1-NS2A-NS2BNS3-NS4A-NS4B-NS5-39. The protein is cleaved into capsid (C), envelope (E), precursor of membrane (prM) and seven non-structural proteins (NS1-NS2A-NS2BNS3-NS4A-NS4B-NS5) (Fig. 2) [30]. The major virion surface protein is E protein. This protein is involved in various features of the viral cycle, membrane fusion and mediating binding. The largest viral protein whose C-terminal portion has RNA-dependent RNA polymerase (RdRP) is NS5 protein activity and its N-terminus is responsible for RNA capping because of its processing due to methyl transferase activity [31]. 428 nucleotides and 27 folding patterns are present in the 39 NCR of the ZIKV genome [30]. These nucleotides and folding patterns may involve in the cyclization, translation, recognition by cellular factors, RNA packaging, recognition by viral factors and genome stabilization [31].

All identified structures of flaviviruses vary on the basis of amino acids that are framing a glycosylation spot in the shell of virus that is composed of two dissimilar proteins having 180 copies. ZIKV varies from other flaviviruses bulges by glycosylation spot on the surface of the virus. A carbohydrate molecule holds numerous sugars tied to the surface of viral protein at this spot. Surrounding residues and glycosylation site on ZIKV may be responsible for attachment of virus to human cells. The amino acids variations among different flaviviruses could suggest the differences in the varieties of human cells where it can attach and infect. If the function of glycosylation spot is analogous to DENV (attachment to the cells receptor of human body) it might be a worthy spot to be targeted by an antiviral compound [32].

Incubation period for ZIKV disease is around 2–7 days [33], with symptoms like influenza syndrome accompanying fever, malaise, headache, dizziness, stomachache, anorexia and maculopapular rash [34]. It can also cause retro orbital eye pain, lymph adenopathy, diarrhea and oedema [15]. Other indications reported are oedema of extremities, gastro intestinal disturbances, photophobia, cough malaise, back pain, aphthous ulcers and sweating. ZIKV infection can be misdiagnosed with other arboviruses and bacterial infections as not explicit to ZIKV infection, especially in prevalent areas. In French Polynesia serious neurological complications with Guillain–Barré syndrome was increased to 20-fold during the epidemic [35].

## Transmission of ZIKV

### ZIKV vector-borne transmission

*Aedes aegypti*, *Aedes polynesiensis* and *Aedes albopictus* are the potential vectors responsible for the transmission of ZIKV infection by biting. *Aedes aegypti* is the foremost vector of DENV and CHIKV. *Aedes polynesiensis* is the main vector responsible for dissemination of lymphatic filariasis in French Polynesia. After the epidemic in French Polynesia these species of mosquitoes were collected and tested for ZIKV infection by RT-PCR and only one *Aedes aegypti* mosquito was confirmed having ZIKV RNA; experimental investigations showed the French Polynesian strain of *Aedes aegypti* can replicate the French Polynesian ZIKV strain (Additional file 1: Figure S1) [36].

Altogether, 61 countries and territories in six WHO regions have confirmation of conventional competent *Aedes aegypti* vectors but have not yet documented ZIKV transmission [37]. Thus, risk of ZIKV spread to other countries is still likely. Might be due to lack of detection fewer countries did not report transmission. The re-emergence or re-introduction was also reported in all areas with prior reports of ZIKV transmission.

Altogether in the African lineage eight mosquitoes were isolated, while P6-740 was the only mosquito isolated in the Asian lineage (Malaysia/1966). In 2007, ZIKV was identified in patients infected with *Aedes aldopictus* mosquitoes from West Africa [14]. However, the *Aedes (stregomyia) hensilli* identified as the probable principal vector that cause Micronesia outbreak [38]. Later on, in 2013, the ZIKV spread out to French Polynesia, with consequent extent to Oceanian islands (New Caledonia, Cook islands, and Easter island), was mostly related with *Aedes aegypti* and *Aedes aldopictus* species [39]. The major symptoms such as rash, low-grade fever, arthralgia, conjunctivitis and GBS (Guillain–Barré syndrome) were observed in 11% of the total population [40]. Furthermore, in Central-South America, *Aedes aegypti* is considered as the utmost common vector for DENV [41]. Later on in 2006, Chouin-Carneiro reported that the New World strains of *Aedes aegypti* and *Aedes albopictus* which found to be poor transmitters of ZIKV results in continuous divergence of the Asian lineage [42]. These strains adopted alternative mode of transmission i.e. direct human to human transmission without the involvement of a vector. Indeed, while *Aedes* is widely accepted as the vector for ZIKV [1, 43, 44], Guedes et al. has revealed that ZIKV can infect and replicate in the salivary glands, midgut, and was also spotted in saliva of *Culex* species [45]. Altogether this work suggests that the transmission vector range for ZIKV may be larger than foreseen (although still a debatable topic demanding more exploration).

### Non-vector-borne transmission

**Fig. 3:**
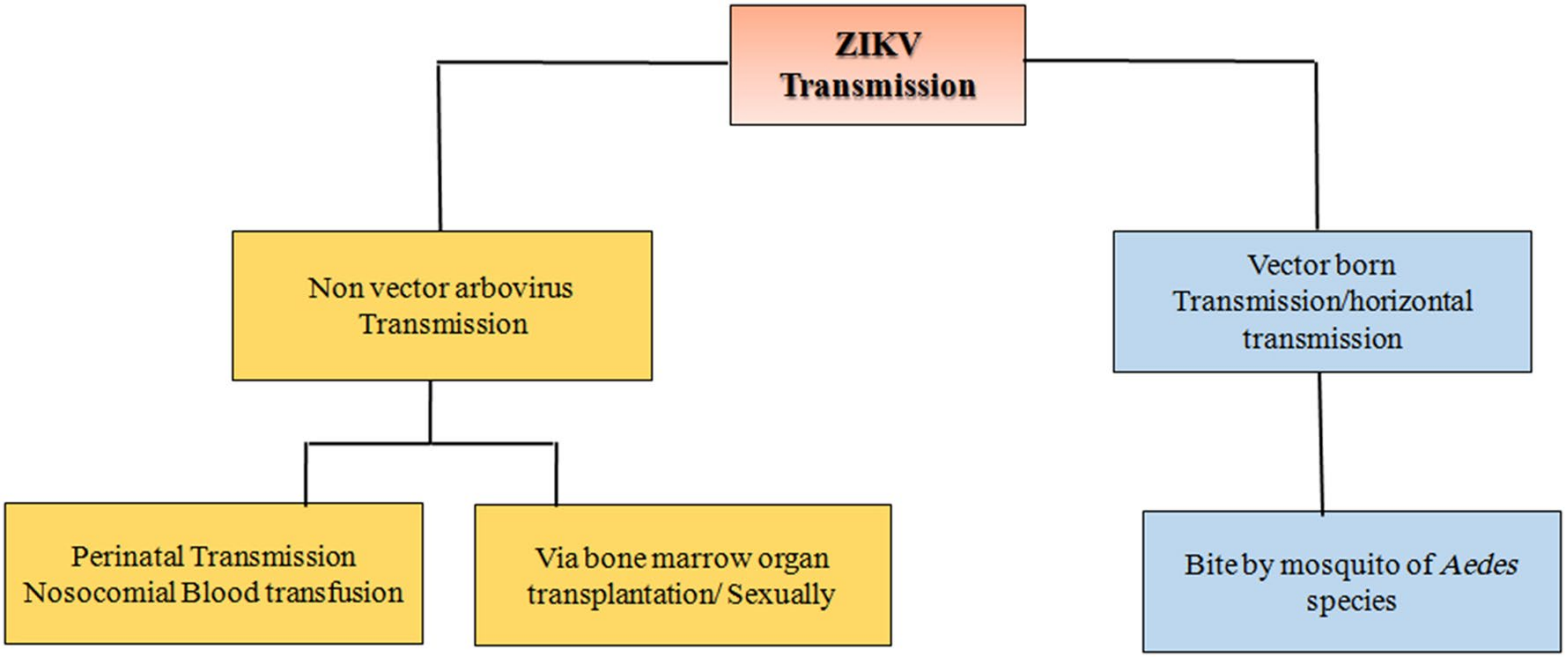
Schematic diagram representing the transmission of ZIKV

Non-vector-borne transmission of ZIKV infection can be caused during labor (mother to child), organ transplantation, blood transfusions and through sexual contact (Fig. 3).

**Fig. 4:**
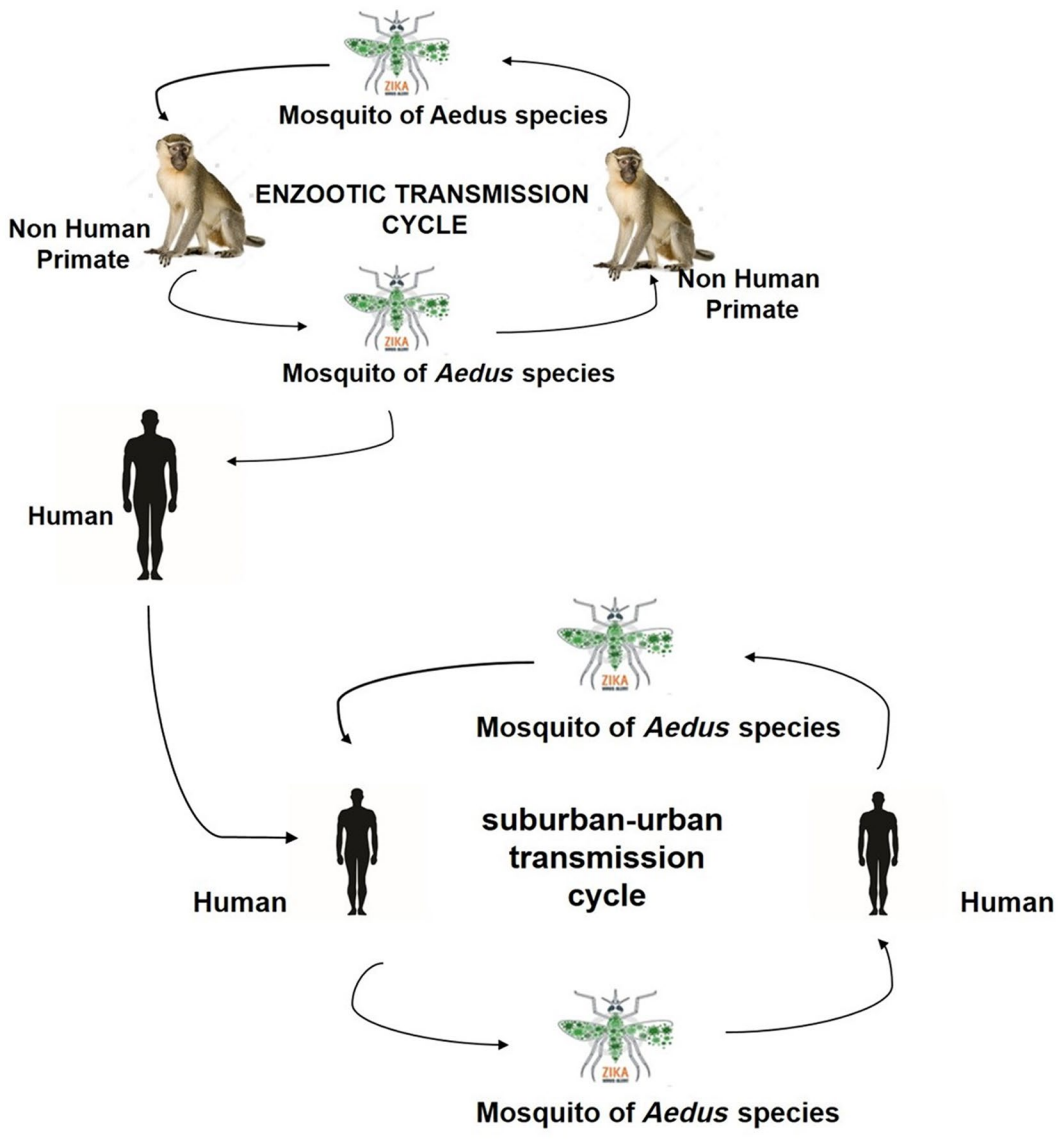
Transmission cycle of ZIKV

Antibodies against ZIKV were detected by Serosurvey studies in goats, rodents (*Meriones hurrianae* and *Tatera indica*), sheep and bats. These studies suggest that there is no clear association between ZIKV and a specific species of animal [36]. In humans, it spreads through the bite of infected *Aedes aegypti* mosquitoes that are usually found in tropical and sub-tropical regions in domestic water-holding containers near dwellings [33]. Consequently, when a mosquito bites a person already infected with ZIKV, the virus infected blood goes into the midgut and prevailed into the circulatory system. Another similar mosquito, *Aedes albopictus* can also transmit ZIKV. Among humans, transmission of this viral infection may also refer to sexual contact [5]. High ZIKV RNA load has detected in breast milk, so transmission is possible by breast feeding and ZIKV can also be transmitted by blood transfusions [46] as reported on December 2015 in Brazil, the first case of ZIKV blood transfusion transmission [47]. ZIKV is adopted to transmit by enzootic and sub Urban cycle (Fig. 4); in enzootic setting this involves mosquitoes of *Aedes* species and non-human primates, however transmission in Urban setting involves human and mosquitoes of *Aedes* species demonstrate vector and non-vector borne transmission of ZIKV.

### Pathophysiology and diagnosis

**Fig. 5:**
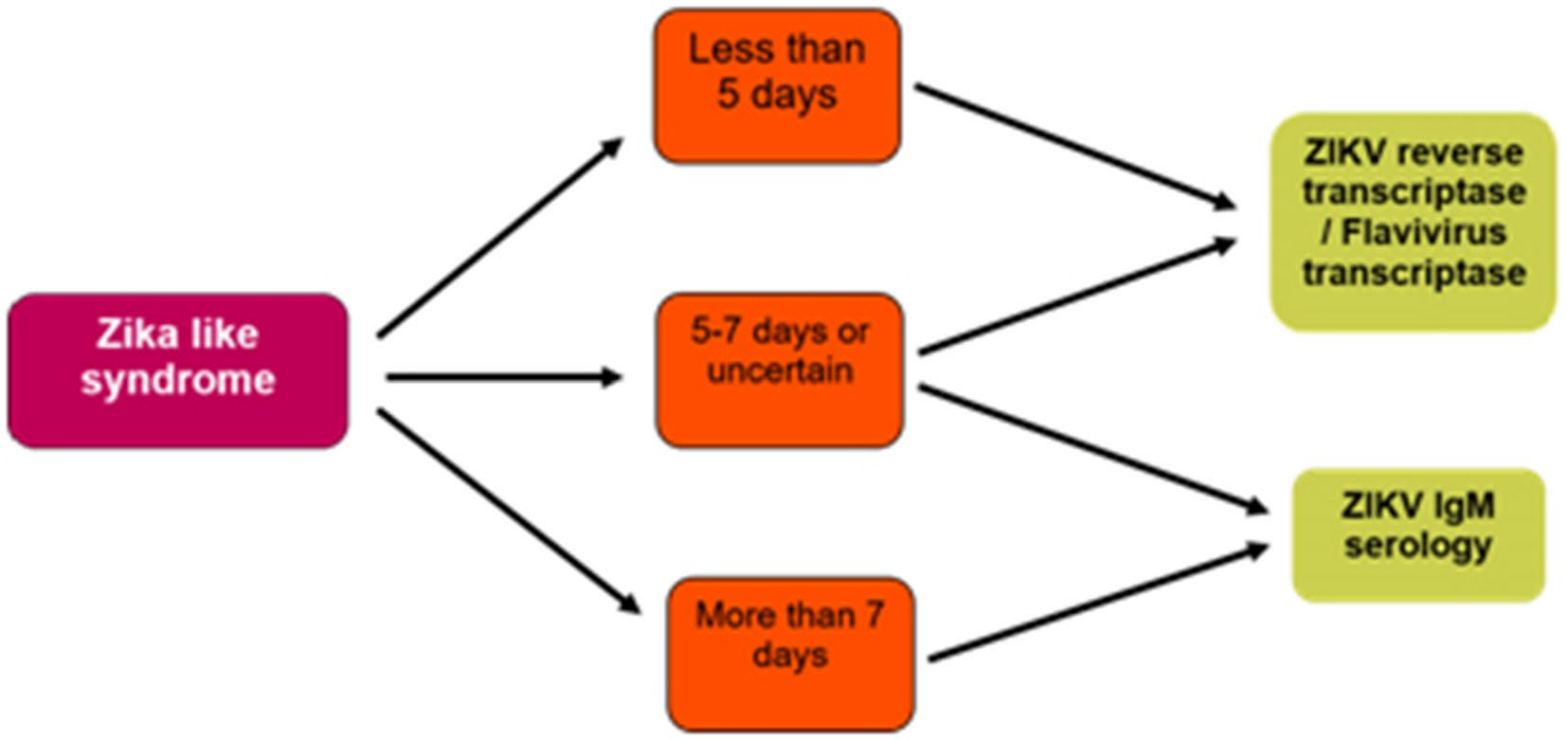
Flow scheme for ZIKV fever diagnosis [73, 74]

In the beginning, ZIKV infection was misdiagnosed with dengue infection. Virus isolation and serological methods are carried out for laboratory diagnosis examination for ZIKV (Table 1) [48]. Virus isolation needs several days (i.e. 1–2 weeks) while convalescent and acute sampling and cross-reactions among flaviviruses are the limitations for serological methods [15]. Cell culture may be utilized to isolate ZIKV [5] but specialized laboratories are required to practice it [5, 16]. Reverse transcription PCR (RT-PCR) is used for confirmation of ZIKV infections whereas IgM against ZIKV can be detected by ELISA [48]. RT-PCR is time saving, specific and sensitive in order to detect ZIKV in serum or cell culture [15]. Molecular detection of ZIKV was increased when saliva was used at the acute phase of disease particularly in children and neonates as blood is difficult to collect [5]. ZIKV fever diagnosis from PAHO is shown in Fig. 5. RT-PCR for ZIKV is done on blood or saliva sample. Sequencing is performed if the results of RT-PCR are positive. ZIKV IgM serology comprises detection by immunofluorescence or ELISA, with confirmation by plaque reduction neutralization test (PRNT) if results are equivocal or positive [36].

**Table 1:** Differential diagnosis of ZIKV infection includes various viral diseases with similar signs and symptoms as ZIKV infection [49]

No	Viral diseases	Similarities with Zika virus	Dissimilarities with Zika virus	Diagnostic test
1	Dengue fever	High fever, severe muscle pain, and headache and may also be associated with hemorrhage	Not associated with conjunctivitis	Serology
2	Chikungunya	High fever and intense joint pain affecting the hands, feet, knees, and back	Not associated with conjunctivitis	Serology
3	Parvovirus	Acute and symmetric arthritis or arthralgia	Rash may or may not be present	Serology
4	Rubella	Low-grade fever, Macular rash, arthritis, lymphadenopathy	Not associated with conjunctivitis, coryza is not present in ZIKV infection	Serology
5	Measles	Fever, cough, conjunctivitis, and lymphadenitis. generalized rash	Sore throat and coryza are not present in ZIKV infection	Serology
6	Leptospirosis	Fever, rigors, myalgia, conjunctival suffusion, headache, arthralgia	Distinguished from ZIKV infection by the presence of jaundice	Serology
7	Malaria	Fever, malaise, nausea, vomiting, abdominal pain, diarrhea, myalgia	Dot associated with conjunctivitis	Visualization of parasites on peripheral smear
8	Rickettsial infection	African tick bite fever and relapsing fever. headache, fever, myalgia, regional lymphadenopathy, generalized rash	Not associated with conjunctivitis	Direct smear and polymerase chain reaction

## Neurological complications of ZIKV infection

Guillain–Barré syndrome and cases of other neurologic manifestations appears in Brazil and French Polynesia throughout ZIKV epidemics, even though it is self-limiting [50, 51]. A report from Ministry of Health of Brazil indicates that there is a possible relation between fetal deformities and infection with ZIKV in pregnancy, as the incidences of microcephaly cases among neonates have amplified by a factor of about 20 [51]. ZIKV infection in fetus can be identified by Ultrasound in second or early third trimester [52]. Approximately 5640 cases of central nervous system malformation and microcephaly have been stated by Brazil comprising 120 deaths in between 22nd October 2015 and 20th February 2016; however, only 163 cases of microcephaly were recorded in Brazil per year on average from 2001 to 2014. Of the 5640 cases, deaths of 120 children occurred during pregnancy or after birth and 30 of these were associated to congenital ZIKV infection [53]. It has been published from the Paraíba State recently that ZIKV infection was detected in newborns having severe congenital CNS malformations. Among these, six cases were reported with confirmed laboratory results of ZIKV; in fetal tissues, amniotic fluid and placenta. All of these reported cases have history of visit to Brazil. There are increasing numbers of cases with intertwined relation between congenital CNS malformations during pregnancy and ZIKV infection [54].

## Risk of Guillain–Barré syndrome

Guillain–Barré syndrome (GBS) is one of the new complications and manifestations of ZIKV infection [34]. GBS is a serious and life threatening neurological disorder eventually resulting in respiratory failure characterized by progressive muscular weakness [55]. Outbreak of ZIKV in French Polynesia four years ago added it to the viruses that can trigger GBS [56]. WHO estimated there could be 3 to 4 million cases of this infection in the following year, therefore, there is a probability of hundreds of cases of GBS. Sufficient intravenous (IV) immunoglobulin (Ig) treatment for patients with ZIKV related GBS should be applied [57]. Increased verification of a ZIKV infection based on laboratory results and GBS prevalence have been reported in 12 countries. In 2015, 1708 GBS cases were recorded in Brazil, indicating a 19% escalation from the preceding year as 1439 GBS cases were reported in 2014. 62% of GBS cases reported in Brazil had a history of signs and symptoms associated to this viral infection. 220 cases of GBS are reported in Colombia while 136 in El Salvador including 5 deaths in the time period from December 2015 to March 2016 [53].

## Treatment of ZIKV

**Fig. 6:**
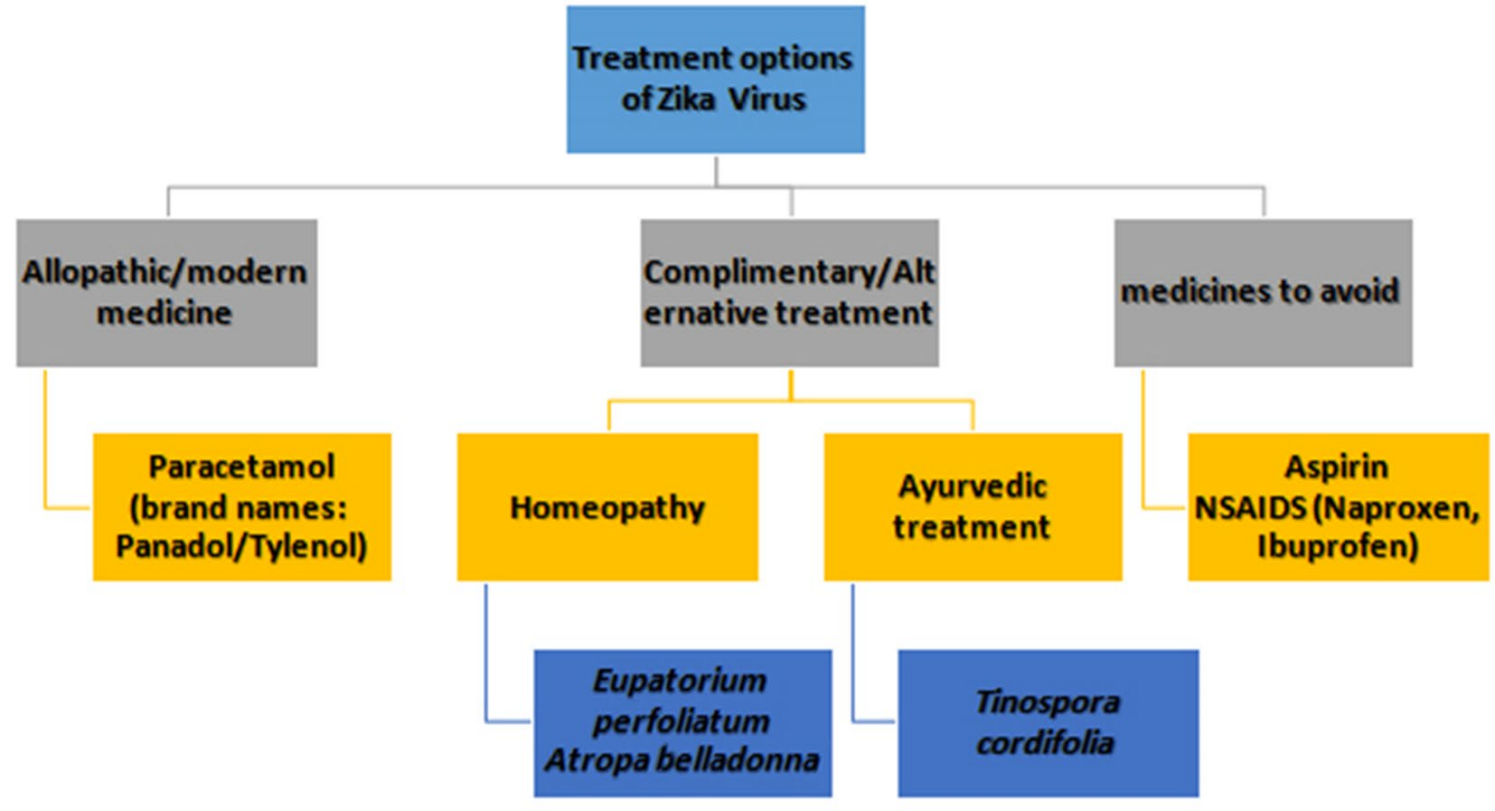
Schematic representation of possible ZIKV treatment

In ZIKV infection, individuals should have adequate water intake, ample rest and treat pain and fever with liquid solutions. If the symptoms aggravate, they should look for counselling and therapeutic consideration (Fig. 6). There are no specific medications or vaccine available to treat or prevent ZIKV infections until now; only medications for symptomatic relief can be considered such as paracetamol to relieve pain and fever associated with this infection [33]. Nonsteroidal anti-inflammatory drugs (NSAIDs) should be avoided and individuals should seek medical advice before taking additional medication if they are already taking medicines for another medical condition [54]. Homeopathy is a worthy treatment option in ZIKV infection as it proved to be effective in Japanese encephalitis virus which is included in the same genus like Zika virus [33]. Treatment with belladonna efficaciously reduced the severity of Japanese encephalitis virus infection [58]. *Atropa belladonna* plant belongs to family Solanaceae [59]. It has been effective in numerous medical conditions having great commercial significance as a major source of alkaloids, mainly scopolamine and hyoscyamine that are pharmaceutical bioactive compounds [60]. Belladonna is native to North Africa, Western Asia and Europe. In *Atropa belladonna* majority of alkaloidal contents are present in ripe fruit and green leaves. It has been used from ancient times in order to treat various human ailments including menstrual disorders, headache, peptic ulcer, inflammation and histaminic reaction [61]. Ultra-diluted belladonna concentrations like 1:10 or 1:100 are used in homeopathy and they are recommended for management of all the infectious diseases and illnesses [62].

Eupatorium is a naturally occurring pharmaceutical homeopathic compound effective against the symptoms of ZIKV disease, so it can be utilized as prophylactic treatment against ZIKV infection [63]. *Eupatorium perfoliatum*, *Rhustox* and *Atropa belladonna* are the homeopathic prescriptions that may be utilized for ZIKV infection treatment. These medicinal agents are effective against the symptoms of ZIKV infection [64]. During epidemics homeopathic pharmaceuticals are more effective in reduction of mortality and morbidity as compare to conventional system of medicines [65].

One of the utmost momentous features of ayurvedic structure is that they are natural substances and free from side effects and there is no scientific evidence of danger for human use [66]. It is a primordial medical science that contains herbal medicines of natural origin with minimal side effects. *Tinospora cordifolia* is a herb and utilized for years as potential immunomodulator and effective natural remedy for viral disease of any nature. It boosts up the immune system and make body resistant enough to fight against infections. Theses herbs potentiate phagocytic abilities of macrophages [67]. Intestinal sickness, urinary tract infections, dengue and swine influenza are effectively treated by the astringent characteristics of these ayurvedic plants so they might also be effective for ZIKV [33].

Beside homeopathic and ayurveda medicines, engineering approaches were also applied to develop peptide therapeutics and support the potential of a brain-penetrating peptide to treat neurotropic viral infections. Therapeutic treatment protected against mortality and evidently lessened symptoms, neuroinflammation and viral loads, furthermore mitigated microgliosis, neurodegeneration and brain damage [68].

Current medical recommendations are directed towards resolving symptoms and not the actual infection; however, ZIKV treatments and vaccines are in development. In 2016, WHO enlist all publicly affirmed commercial, government and academic-led projects focused at ZIKV interventions, together with vaccines [69]. The list encompasses numerous approaches, comprising vaccines via purified inactivated virus, Virus-like particles (VLP), protein subunits, DNA and live recombinant attenuated viruses. Since April 2019, no vaccines have been permitted for clinical usage, though utmost were in the clinical stages of development [70, 71].

**Fig. 7:**
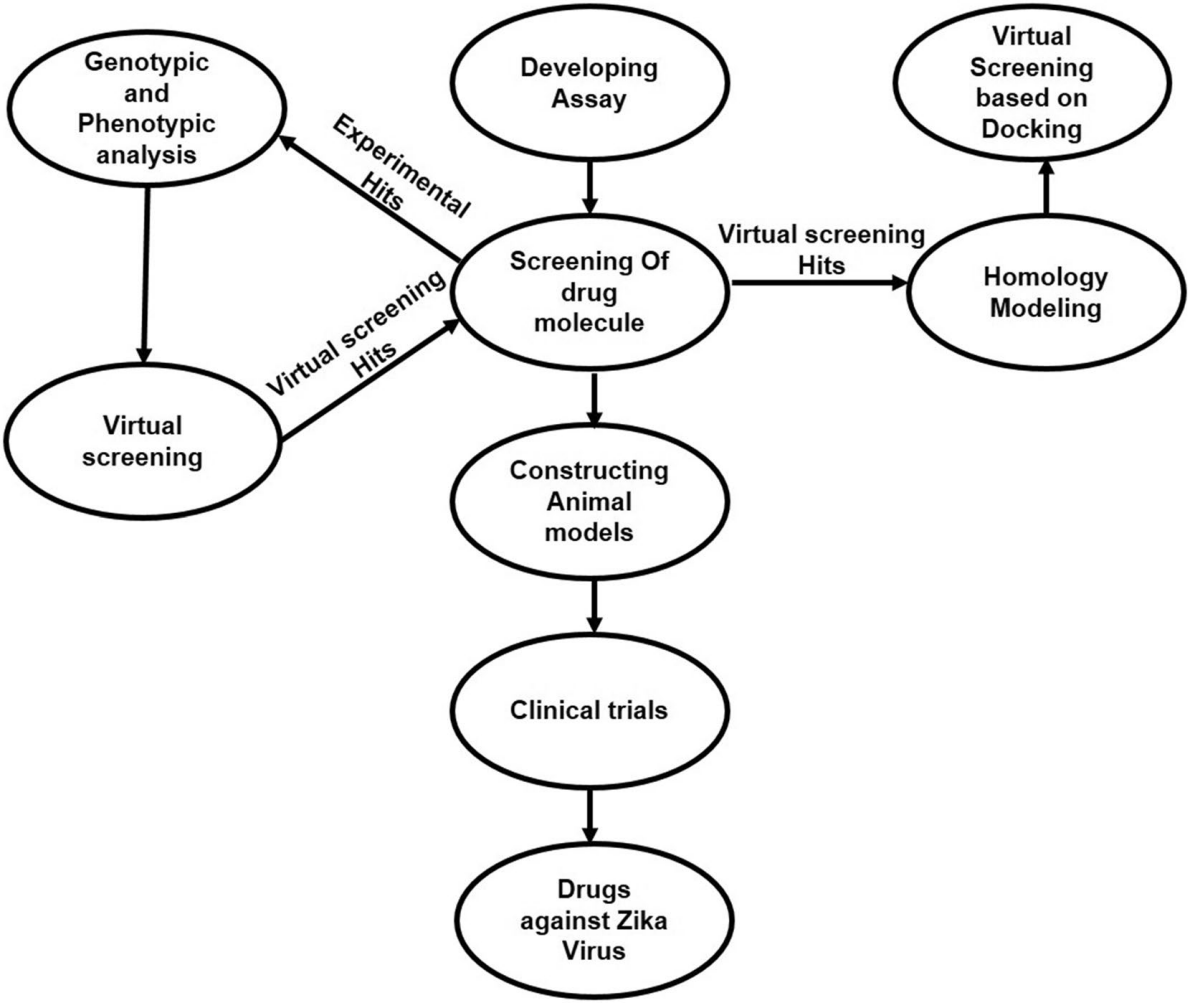
Proposed workflow for drug development against ZIKV

Suggested workflow for prompt discovery of drug counter to ZIKV is presented in Fig. 7; whole process is proposed to initiate from screening moderate or high-throughput in vitro analysis development following with testing of approved drugs or other antiviral agents. Virtual screening based on docking could be selected for testing further compounds by means of advanced model of homology or phenotypic and genotypic analyses if drug repurposing will be unsuccessful. Priority can be given to the compounds resulting from docking for in vitro analysis in parallel. Consequent steps are typical as a pipeline in the discovery of any drug including developing the models of animals, clinical trial and if getting optimistic results, manufacturing the drug against ZIKV by scale up process, advertising and dissemination of drug [72].

## Prevention and control of ZIKV

Most precarious threats for ZIKV infection are mosquitoes including their reproducing localities. Their encounter with humans must be reduced in order to control and prevent their outspread. This can be employed by using mosquito repellents, mosquito nettings and closing the entrances and openings. Insect killing sprays recommended by the WHO Pesticide Evaluation Scheme should be used as larvicides [27, 34]. Insect repellents should not be used for babies under two months, mosquito nets should be used to protect babies from insect bite. Centre of disease control recommends mosquito repellents with active ingredients picaridin, DEET, eucalyptus oil, IR3535, oil of lemon and para-menthane-diol. These are safe for pregnant and lactating mothers [54]. Repellants containing eucalyptus oil, lemon oil and para-menthane-diol should be avoided for children below 3 years of age. Mosquitoes should be killed using indoor mosquito killing sprays which contain active ingredient Imidacloprid and β-Cyfluthrin available in market [75]. Flying insect fogger can also be used against the mosquitoes containing active ingredients Tetramethrin and Cypermethrin [75]. Tests against ZIKV infection should be performed before blood transfusions to prevent transfusion related transmission. Pregnancy must be avoided in the high risk ZIKV infection prone areas before complete eradication or extra care must be exercised as microcephaly is associated with ZIKV infection [76].

Besides, different vector control strategies for averting Zika virus spread can be employed. Subjugation of mosquito population can be accomplished by a bacterium that can infect mosquitoes. Other strategies include the use of intracellular bacteria *Wolbachia*, which acts as a biopesticide to control mosquito population. Larvae of *Toxorhynchites splendens* mosquito species does not feed on blood. They feed on the larvae of other mosquito species, while the adults feed on honeydew, fruit, and nectar [77]. Hence, the spread of ZIKV can be encountered by utilizing these species. *Aedes* species mosquitoes populations can also be suppressed by the strategy of using sterile males to induce sterility in wild fertile females [78].

## Future directions

Mosquito-borne epidemics are critically aggravating the pre-existing burden that the primary healthcare systems face. Work force will be affected and the societies may be threatened by the epidemic wave if they are not prepared well. Improved investigation and actions against response are required to alleviate the substantial burden on health systems and control promoting it worldwide. At present there is no vaccine available for ZIKV infection. Vaccines against flaviviral infections available for use of human are yellow fever vaccine, Japanese encephalitis vaccine, tick-borne encephalitis vaccines and dengue vaccine, so the rules for the vector borne infections must be followed in order to prevent ZIKV infection, as well as avoiding mosquito bite and control of vector is the only available options. Animal models of the ZIKV disease are immediately required not only for exhibiting the materno-fetal transmission and confirmation of its neurologic manifestations but also to report the influence of virus on host’s immunity and reproductive health throughout the life. ZIKV infection is increasing dramatically, so it is the need of hour to take some necessary steps to eliminate this lethal infection and to constrain its future entrance as well. ZIKV specific rapid molecular diagnosis should be done urgently in order to detect the infection in less time before it aggravates. Modern techniques of molecular biology should be utilized to make vaccine specific to ZIKV. Research gaps should be addressed promptly and systematically. This can be accomplished by understanding the occurrence of broad spectrum clinical consequences that are resulting from fetal ZIKV infection and the environmental influences that effect their emergence. This also require the advancement of flaviviruses selective investigative tools, models of animals to detect developing effects of fetus resulting from viral septicity [79, 80], novel products to control vector and strategies, effective medications and the vaccines to shield humans counter to ZIKV disease.

## Supplementary information

**Additional file 1: Figure S1:** Reproductive cycle of ZIKV.

## Data Availability

Not applicable.
