# Supplementary figures and images for: A review on Zika virus outbreak, epidemiology, transmission and infection dynamics
Source: J Biol Res (Thessalon). 2020 Mar 4;27:5. doi: 10.1186/s40709-020-00115-4 (PMC7057477; doi:10.1186/s40709-020-00115-4)

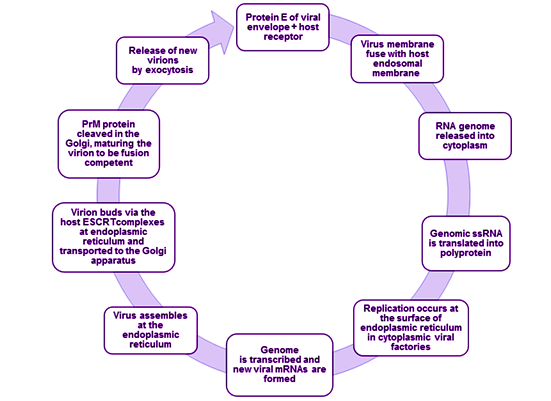

Supplement: Supplementary file 1 — Additional file 1: Figure S1: Reproductive cycle of ZIKV. [file 40709_2020_115_MOESM1_ESM.tif]
